# Primary Central Nervous System Lymphoma Presenting As Chronic Subdural Hematoma: Case Report and Review of the Literature

**DOI:** 10.7759/cureus.7043

**Published:** 2020-02-19

**Authors:** Alexa Semonche, Pablo Gomez, John Paul G Kolcun, Roberto J Perez-Roman, Robert M Starke

**Affiliations:** 1 Neurological Surgery, University of Miami Miller School of Medicine, Miami, USA; 2 Neurological Surgery, Rutgers Robert Wood Johnson Medical School, New Brunswick, USA; 3 College of Arts, Sciences, and Education, Florida International University, Miami, USA; 4 Neurological Surgery, Rush University Medical Center, Chicago, USA

**Keywords:** lymphoma, primary central nervous system lymphoma, chronic subdural hematoma, neuroimaging, differential diagnosis

## Abstract

Primary central nervous system lymphoma (PCNSL) rarely manifests in immunocompetent patients. In such cases, these lesions may mimic more common intracranial bleeding or tumors. We present the case of an elderly patient who presented with a presumed chronic subdural hematoma (SDH); upon surgical intervention, an occult mass was discovered with no evidence of associated hematoma. Biopsy and immunohistochemistry demonstrated PCNSL. Literature review identified six other cases of PCNSL in immunocompetent adults that were initially suspected to be SDH but were finally diagnosed with PCNSL. Our literature review highlights the rarity these cases and the importance of distinguishing intracranial bleeds from PCNSL, as the latter can be treated with chemoradiation with good clinical outcomes.

## Introduction

Primary central nervous system lymphoma (PCNSL) is a rare variant of non-­Hodgkin’s lymphoma (NHL), which is restricted to the brain, leptomeninges, cranial nerves, spinal cord, or intraocular compartment without involvement of other organ systems. PCNSL comprises 1%-3% of all central nervous system (CNS) tumors and only 2%-3% of NHL cases [1]. In contrast, secondary CNS lymphoma (SCNSL) represents disseminated systemic NHL and affects up to 27% of NHL patients [2].

The primary risk factor for PCNSL is immunodeficiency, most commonly affecting patients with AIDS and low (<100,000) CD4 count [3]. However, the success of highly active antiretroviral therapy and increasing demographic of elderly patients have been associated with decreasing incidence of PCNSL in immunocompromised patients and increasing rates in elderly immunocompetent patients, especially those >65 years of age [4]. In immunocompetent patients, PCNSL presents at a median age of 60 years with a slight sex predilection for males (1.2:1 male:female) [4].

Without treatment, the prognosis for PCNSL is dismal (median survival of 1.5 months after diagnosis). Standard-of-care therapy with high-dose methotrexate (MTX) in combination with other chemotherapy agents (e.g. rituximab, cytarabine) followed by whole brain radiation greatly improves overall survival up to a median of three years [3]. The prognosis for SCNSL remains poor at 2.2 months after diagnosis, although this may be improved with prophylactic MTX chemotherapy [3].

Given that chemoradiation can meaningfully improve overall survival in PCNSL, it is important for practitioners to recognize this pathology in patients without a history of immunodeficiency or NHL. PCNSL often manifests clinically with nonspecific signs (e.g. headache, focal neurological deficits, altered mental status) [5]. Therefore, a critical step in diagnostic workup is recognizing suggestive features of PCNSL on neuroimaging studies. T1-weighted magnetic resonance imaging (MRI) is the highest-yield study according to recent consensus guidelines; PCNSL is typically hypointense and homogeneously enhancing, although up to 13% of lesions can have a ring-enhancing appearance [6]. Parenchymal PCNSL lesions are typically periventricular, but can also be centrally located in the frontal lobes, basal ganglia, or cerebellum [7]. In contrast, primary dural-based CNS lymphomas arise from the leptomeningeal space. SCNSL can present with similar radiographic findings and is commonly accompanied by leptomeningeal enhancement [7]. Definitive diagnosis of PCNSL requires histopathological confirmation via stereotactic biopsy with immunohistochemical staining for NHL subtype markers [6].

Although suggestive features of PCNSL on neuroimaging have been identified, they can mimic other pathologies that require different neurosurgical intervention. To illustrate this point, we present the case of an immunocompetent patient with presumed chronic subdural hematoma (SDH) which was ultimately revealed to be a PCNSL on histopathological diagnosis. We then review the literature for cases of PCNSL or SCNSL mimicking other pathologies on neuroimaging to bring them to the attention of the neurosurgical community.

## Case presentation

**Figure 1:**
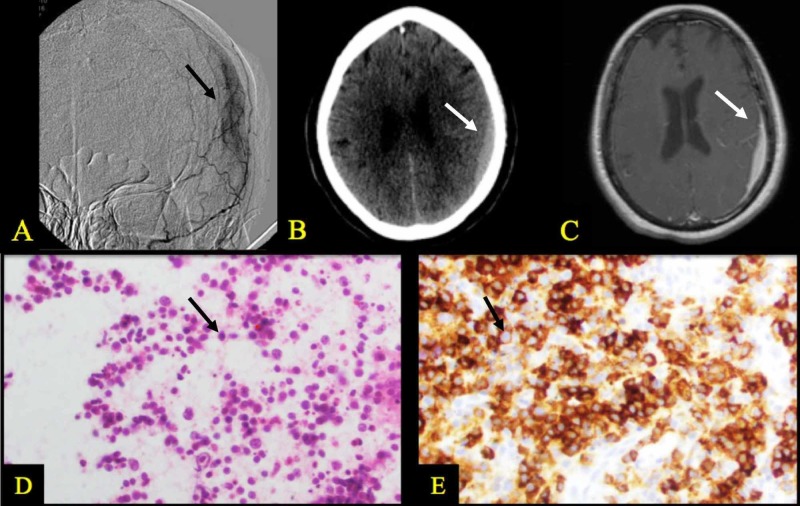
Imaging and histopathology findings demonstrating primary central nervous system lymphoma presenting as chronic subdural hematoma (A) Left external carotid artery angiogram, demonstrating arterial phase blush within the left convexity lesion. (B) CT brain without contrast, demonstrating a left parietal extra-axial collection, hyperdense to surrounding brain. (C) MRI brain with gadolinium contrast, demonstrating a homogeneously enhancing extra-axial lesion of the left parietal region. (D) HIstopathological results from biopsy of subdural lesion, demonstrating numerous neoplastic B cells with variably enlarged atypical nuclei. (E) Biopsy of subdural lesion, demonstrating expression of the B-cell marker CD20 by immunohistochemical staining.

A 63-year-old woman presented to our neurosurgical service one year after a fall with head impact. In the intervening year since her fall, she had multiple admissions at outside hospitals for episodic confusion, vomiting, and generalized tonic­-clonic seizures. Computed tomography (CT) imaging during this time demonstrated what appeared to be a small subacute SDH. On presentation to our service, the patient had a nonfocal neurological exam and her clinical symptoms were resolving. Given the hematoma’s persistence after the fall, a cerebral angiogram was performed to rule out dural arteriovenous malformation (Figure 1A). Although the angiogram demonstrated a slight arterial blush in the area of the hematoma, extensive imaging revealed no evidence of an obvious fistula. She was subsequently followed with serial imaging for more than two years, which demonstrated interval growth of the frontoparietal lesion from 8 to 15 mm for more than one year (Figure 1B). Further MRI showed homogenous enhancement of the suspected subacute SDH (Figure 1C). As a result, the patient was taken to the operating room for a craniotomy to evacuate the hematoma. Upon opening the dura, however, an occult lesion was identified with no evidence of hematoma. Multiple samples were sent for pathology, which demonstrated low­-grade B­-cell lymphoma (Figure 1D, 1E). Extensive work­up including bone marrow biopsy and CT of the chest, abdomen, and pelvis revealed no signs of disease elsewhere. HIV antigen testing was negative. Postoperatively, the patient did well, and was referred to medical and radiation oncology services to discuss chemoradiation therapy.

## Discussion

This case demonstrates a rare case of PCNSL in an immunocompetent patient mimicking a chronic SDH without evidence of hematoma on neurosurgical intervention. We then performed a literature review of all similar cases of PCNSL in immunocompetent adults with an initial diagnosis of an intracranial bleed based on radiographic findings, no finding of hematoma on surgical intervention, and final diagnosis of PCNSL based on histopathological findings.

Our review of the literature identified five articles describing six cases, making this an extremely rare presentation of PCNSL (Table 1) [8-12]. The six patients were all women aged 39-62 years at time of diagnosis. In all cases, the initial suspected diagnosis was SDH; in five of the six cases, the final diagnosis was low-grade dural-based primary B-cell mucosa-associated lymphoid tissue (MALT) lymphoma. The remaining case was a dural primary diffuse large B-cell lymphoma (DLBCL). The dural extension of the lymphoma is these cases is consistent with the subdural/convexity location of the lesion on imaging. The predominance of low-grade B-cell lymphoma is also consistent with previous studies reporting an association between dural-based CNS lymphoma and low-grade B-cell lymphoma; in contrast, parenchymal CNS lymphoma more commonly represents the more aggressive DLBCL subtype [13]. In all cases, extensive diagnostic workup for extra-CNS lymphoma involvement was negative. Once PCNSL was correctly diagnosed and managed with radiotherapy and/or chemotherapy, clinical outcomes for the patients with low-grade MALT lymphoma were generally good, with no recurrence noted at final follow-up. The only case of rapid neurological deterioration and death following diagnosis occurred in the patient with high-grade DLBCL.

**Table 1:** Literature review of PCNSL cases mimicking intracranial bleeds with no evidence of hematoma on surgical intervention. DLBCL = diffuse large B-cell lymphoma, F = female, hx = history, HA= headache, ICP = intracranial pressure, L = left, MALT = mucosa-associated lymphoid tissue, MTX = methotrexate, PCNSL = primary central nervous system lymphoma, R = right, RT = radiotherapy, SAH = subarachnoid hemorrhage, SDH = subdural hematoma, Sx = symptoms, T1W = T1-weighted, T2W = T2-weighted, WBRT = whole brain radiation therapy.

Authors, year	Age, sex	Presenting Sx	Imaging findings	Initial diagnosis	Initial management	Final diagnosis	Final management; clinical outcome
Kambham et al., 1998 [8]	39F	Weakness, hearing loss	MRI: L cerebellopontine angle lesion	Meningioma vs. SDH	Craniotomy with subtotal resection; biopsy	PCNSL-MALT lymphoma	RT; Alive at four years
	62F	HA	MRI: L parieto-occipital dural-based lesion	Meningioma vs. SDH	Biopsy	PCNSL-MALT lymphoma	RT: alive at six months
Goetz et al., 2002 [9]	64F	HA, sudden-onset L hemiparesis	Initial presentation: CT: R frontoparietal hyperdense mass; recurrent presentation: T1W MRI: hypointense R frontoparietal dural-based convexity lesion; T2W MRI: hyperintense	Initial presentation: SDH; recurrent presentation: meningioma	Initial presentation: low-dose dexamethasone; Recurrent presentation: craniotomy, no hematoma found; excision of mass	PCNSL-MALT lymphoma	WBRT; no recurrence at three months
Gocmen et al., 2010 [10]	45F	Six-month hx of seizures, speech disturbances	CT: enhancing L frontotemporal mass with midline shift; T1W MRI: isointense lesion; T2W MRI: hypointense lesion	SDH	Craniotomy, no hematoma found; biopsy of thickened dura encountered intraoperatively	PCNSL-MALT lymphoma	Chemotherapy; no recurrence at last follow-up
Sacho et al., 2010 [11]	46F	Six-week hx of HA, sudden-onset neurological decline, seizures	CT: R parietal contrast-enhancing subdural mass	SDH vs. meningioma vs. empyema	Urgent craniotomy; no hematoma found; subtotal resection of mass	PCNSL-DLBCL	MTX chemotherapy; salvage craniotomy, neurological deterioration and death six weeks postop from elevated ICP
Jesionek-Kupnicka et al., 2013 [12]	60F	Three-week hx of HA, R upper extremity weakness, R facial cramping	T1W MRI: L pareito-occipital isointense mass; T2W MRI: hypointense	SDH with traumatic SAH	Urgent craniotomy, no hematoma found; biopsy of tumor mass encountered intraoperatively	PSCNL-marginal zone MALT lymphoma	RT; no recurrence at last follow-up

Although our literature review focuses on immunocompetent patients with PCNSL, we recognize that SCNSL can also present like a subdural bleed in patients with a history of B-cell lymphoma. Gonzalez-Bonet et al. described the case of a 71-year-old male who presented with a two-week history of neurological decline after a fall with head impact [14]. Although CT imaging was suspicious for bilateral SDH, the lesion was found to be recurrent mantle B-cell lymphoma on histopathology. Bone marrow biopsy confirmed the recurrence of systemic lymphoma. The patient responded well to a three-month course of ibrutinab chemotherapy and remains recurrence-free at six-month follow-up.

Another clinical entity to consider is PCNSL presenting with intracranial hemorrhage, which can obscure the lymphoma tumor on neuroimaging. Barrios et al. described the case of a 40-year-old woman who experienced sudden-onset neurological deterioration and was found to have a SDH from a ruptured dural lymphoma lesion on autopsy, along with multiorgan dissemination of small B-cell lymphoma [15]. Although this case does not describe a case of CNS lymphoma mimicking another diagnosis on neuroimaging, it does illustrate how CNS lymphoma and an intracranial bleed are not mutually exclusive pathologies. Similarly, Alimehmeti and Locatelli reported the case of an 85-year-old woman who presented with progressive drowsiness, aphasia, and tetraparesis whose imaging was concerning for a chronic SDH [16]. Urgent decompressive craniotomy for hematoma evacuation revealed an underlying tumor mass, which was found to be a B-cell lymphoma. Two other cases by Reyes et al. and Gotoh et al. reported similar phenomena of chronic SDH associated with a malignant B-cell lymphoma mass [17,18]. Only the two previous cases, reported by Kim et al. and Rubenstein et al., have described parenchymal PCNSL lesions associated with intracerebral hemorrhage [19,20].

## Conclusions

PCNSL in immunocompetent patients remains an uncommon diagnosis. As there is low clinical suspicion for such cases, these lesions can easily mimic more typical entities. We present the rare case of a patient with long-standing PCNSL whose clinical course and imaging studies mimicked SDH for several years. Our patient and six others identified via literature review demonstrate how correct diagnosis and appropriate management of PCNSL can result in good clinical outcomes, especially in those with low-grade B-cell lymphomas. We further consider PCNSL presenting with concomitant intracranial bleed, SCNSL mimicking SDH, and other types of intracranial tumors that PCNSL can mimic. Our findings illustrate the rarity of PCNSL mimicking intracranial bleed and the importance of obtaining histopathological diagnosis in these cases.
